# Mga*Spn* and H-NS: Two Unrelated Global Regulators with Similar DNA-Binding Properties

**DOI:** 10.3389/fmolb.2016.00060

**Published:** 2016-09-29

**Authors:** Virtu Solano-Collado, Mário Hüttener, Manuel Espinosa, Antonio Juárez, Alicia Bravo

**Affiliations:** ^1^Centro de Investigaciones Biológicas, Consejo Superior de Investigaciones CientíficasMadrid, Spain; ^2^Departament de Microbiologia, Facultat de Biologia, Universitat de BarcelonaBarcelona, Spain; ^3^Institut de Bioenginyeria de CatalunyaBarcelona, Spain

**Keywords:** global transcriptional regulators, nucleoid-associated proteins, Mga/AtxA family, protein-DNA interactions, DNA bendability

## Abstract

Global regulators play an essential role in the adaptation of bacterial cells to specific niches. Bacterial pathogens thriving in the tissues and organs of their eukaryotic hosts are a well-studied example. Some of the proteins that recognize local DNA structures rather than specific nucleotide sequences act as global modulators in many bacteria, both Gram-negative and -positive. To this class of regulators belong the H-NS-like proteins, mainly identified in γ-Proteobacteria, and the Mga*Spn*-like proteins identified in Firmicutes. H-NS and Mga*Spn* from *Escherichia coli* and *Streptococcus pneumoniae*, respectively, neither have sequence similarity nor share structural domains. Nevertheless, they display common features in their interaction with DNA, namely: (i) they bind to DNA in a non-sequence-specific manner, (ii) they have a preference for intrinsically curved DNA regions, and (iii) they are able to form multimeric complexes on linear DNA. Using DNA fragments from the hemolysin operon regulatory region of the *E. coli* plasmid pHly152, we show in this work that Mga*Spn* is able to recognize particular regions on extended H-NS binding sites. Such regions are either located at or flanked by regions of potential bendability. Moreover, we show that the regulatory region of the pneumococcal *P1623B* promoter, which is recognized by Mga*Spn*, contains DNA motifs that are recognized by H-NS. These motifs are adjacent to regions of potential bendability. Our results suggest that both regulatory proteins recognize similar structural characteristics of DNA.

## Introduction

Global modulators play key roles in the ability of bacterial cells to rapidly adapt to environmental fluctuations by adjusting their gene expression pattern. As a consequence, they enable the pathogenic bacteria to colonize and survive in different niches of their eukaryotic hosts. Whereas, some global modulators recognize specific DNA sequences, others exhibit a preference for particular DNA structures. Examples of the latter group are the H-NS-like proteins, mainly found in γ-Proteobacteria, and the Mga*Spn*-like proteins identified in Firmicutes.

In *Escherichia* and *Salmonella*, the DNA-binding properties of the nucleoid-associated protein H-NS (137 amino acids) have been studied in detail (for a review see Winardhi et al., 2015). Like other nucleoid-associated proteins, H-NS is involved in both organization of the bacterial chromosome and regulation of gene expression. H-NS functions generally as a repressor or gene silencer, but it can also act indirectly as a transcriptional activator (Ko and Park, 2000). Many genes regulated by H-NS encode virulence determinants. H-NS consists of an N-terminal oligomerization domain and a C-terminal DNA-binding domain. In solution, H-NS is able to form higher-order oligomers. This ability correlates with its ability to form nucleoprotein filaments (Lim et al., 2012). Imaging studies have revealed that H-NS-like proteins are able to organize large DNA molecules into various conformations, including extended nucleoprotein filaments, hairpin-like large DNA bridges, and higher-order DNA condensations (Dame et al., 2000; Liu et al., 2010).

*In vitro* DNA binding experiments have shown that H-NS binds to DNA in a non-specific manner, although it has a strong preference for intrinsically curved AT-rich DNA regions. Furthermore, high-affinity DNA-binding sites for H-NS have been identified in AT-rich regions of the chromosomal DNA (Lang et al., 2007). Formation of H-NS nucleoprotein filaments from such high-affinity sites (nucleation sites) may lead to selective gene silencing either by inhibiting the binding of the RNA polymerase to the promoter region or by blocking RNA polymerase translocation (reviewed by Winardhi et al., 2015). Studies on the *LEE5* promoter supported an additional model for H-NS-mediated repression. In this model, H-NS spreading from a site located upstream of the *LEE5* promoter to a site located at the promoter would facilitate specific contacts between H-NS and the RNA polymerase (Shin et al., 2012).

**Figure 1:**
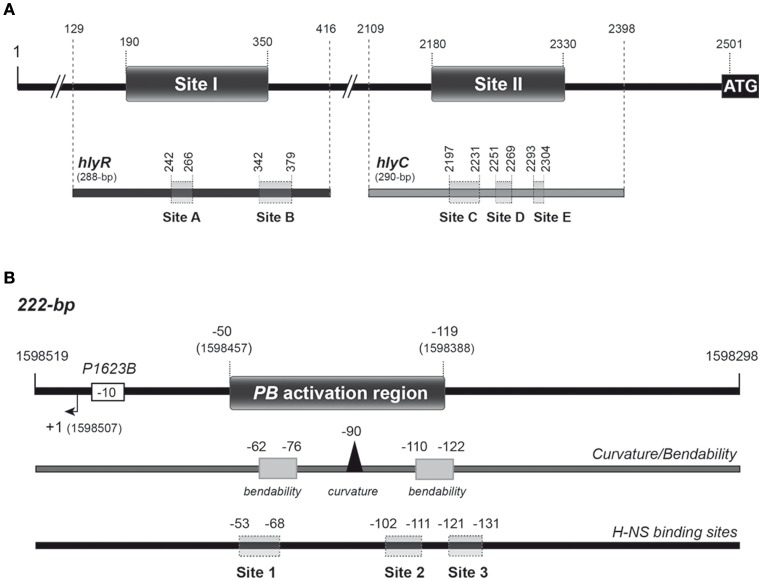
**Relevant features of the DNA fragments used as targets for H-NS-His and Mga***Spn***-His in this study. (A)** The *hly* regulatory region of plasmid pHly152. The location of the two H-NS binding sites (sites I and II) defined by DNase I footprinting assays (Madrid et al., 2002) is indicated. Both sites are located upstream of the *hlyC* gene, the first gene of the *hly* operon. The ATG translation start codon of *hlyC* (coordinate 2501) and the coordinates of the *hlyR* (288-bp) and *hlyC* (290-bp) DNA fragments are shown in the upper part of the Figure. Shadowed boxes on the *hlyR* and *hlyC* DNA fragments (lower part of the Figure) represent the Mga*Spn*-His binding sites (sites A, B, C, D, and E) defined by DNase I footprinting assays in this study. **(B)** The pneumococcal 222-bp DNA fragment. It corresponds to the region spanning coordinates 1598298 and 1598519 of the R6 chromosome. The *P1623B* promoter and the corresponding transcription start site (+1) are indicated. This fragment contains the *PB* activation region (positions −50 to −119 relative to the transcription start site of the *P1623B* promoter) (Solano-Collado et al., 2012). The *PB* activation region contains a peak of potential intrinsic curvature (arrowhead) that is flanked by regions of potential bendability (gray boxes) (Solano-Collado et al., 2013). The sites (sites 1, 2, and 3) recognized preferentially by H-NS-His on the 222-bp DNA fragment are shown (this work).

H-NS is known to modulate the expression of the thermoregulated hemolysin (*hly*) operon (genes *hlyC, hlyA, hlyB*, and *hlyD*), which encodes the toxin α-hemolysin and additional gene products required for its activation and export. This toxin is produced by several uropathogenic *E. coli* strains. The *hly* operon of the *E. coli* plasmid pHly152 has been studied in detail. First, Vogel et al. (1988) identified an essential regulatory sequence located ~2 kbp upstream of the *hly* operon, the so-called *hlyR* sequence. Between the *hlyR* sequence and the promoter region of the *hly* operon there is an IS*2* insertion element. Subsequently, Madrid et al. (2002) identified two extended H-NS binding sites upstream of the *hly* operon (see Figure 1A). One of them (site I; nucleotides 190 to 350 of plasmid pHly152) is located within the *hlyR* sequence. The second one (site II; nucleotides 2180 to 2330) overlaps the promoter region of the *hly* operon. A deletion analysis confirmed the relevance of site I for thermoregulation of the *hly* operon (Madrid et al., 2002).

The Mga*Spn* transcriptional regulator (493 amino acids) contributes to the virulence of *Streptococcus pneumoniae*. Some DNA-binding properties of Mga*Spn* resemble the ones reported for H-NS. Specifically, *in vitro* DNA binding studies (gel retardation, footprinting and electron microscopy) have shown that Mga*Spn* generates multimeric complexes on linear double-stranded DNA. Furthermore, Mga*Spn* binds to DNA with little or no sequence specificity, and shows a preference for DNA regions that contain a potential intrinsic curvature (Solano-Collado et al., 2013). Nevertheless, despite this fact, Mga*Spn* and H-NS are unrelated proteins. They neither have sequence similarity nor share structural domains. Mga*Spn* is a member of a new class of global response regulators known as the Mga/AtxA family, which includes the Mga, AtxA and MafR proteins from *S. pyogenes, Bacillus anthracis* and *Enterococcus faecalis*, respectively (Hondorp et al., 2013; Hammerstrom et al., 2015; Ruiz-Cruz et al., 2016). According to the Pfam database (Finn et al., 2016), Mga*Spn* has two putative N-terminal DNA-binding domains, the so-called HTH_Mga (residues 6 to 65) and Mga (residues 71 to 158) domains (Solano-Collado et al., 2012). These domains do not exhibit sequence similarity with the C-terminal DNA-binding domain of H-NS.

Mga*Spn* plays a significant role in both nasopharyngeal colonization and lung infection in mice (Hemsley et al., 2003). *In vivo* experiments showed that Mga*Spn* activates the pneumococcal *P1623B* promoter and, consequently, the transcription of a four-gene operon (*spr1623-spr1626*) of unknown function. This activation requires a 70-bp region (*PB* activation region) located upstream of the *P1623B* promoter (Solano-Collado et al., 2012) (see Figure 1B). Interestingly, Mga*Spn* recognizes the *PB* activation region as a primary binding site when it is located at internal position on a 222-bp DNA fragment, but not when it is positioned at one end of the DNA fragment (Solano-Collado et al., 2013). According to the bend.it program (Vlahovicek et al., 2003), the *PB* activation region contains a potential intrinsic curvature flanked by regions of bendability (Figure 1B).

From the information mentioned above, it is apparent that H-NS and Mga*Spn* share some DNA-binding properties. In light of these observations, we hypothesized that both unrelated proteins might recognize similar characteristics of DNA. In this work we present evidence that supports this hypothesis. By gel retardation and DNase I footprinting assays, we show that Mga*Spn* is able to recognize particular regions on extended H-NS binding sites and vice versa.

## Materials and methods

### Polymerase chain reaction (PCR)

The Phusion High-Fidelity DNA polymerase (Thermo Scientific) was used. Reaction mixtures (50 μl) contained 5–20 ng of template DNA, 20 pmol of each primer, 200 μM each deoxynucleoside triphosphate (dNTP), and one unit of DNA polymerase. A initial denaturation step was performed at 98°C for 1 min, followed by 30 cycles that included the next steps: (i) denaturation at 98°C for 10 s, (ii) annealing of the primers around 55°C (depending on the primer melting temperature) for 20–30 s, and (iii) extension at 72°C for 20–40 s (depending on the amplicon length). A final extension step was performed at 72°C for 10 min. PCR products were cleaned up with the QIAquick PCR purification kit (Qiagen).

### PCR-amplification of DNA regions

Oligonucleotides used for PCR amplifications are listed in Table 1. As DNA templates, chromosomal DNA from the pneumococcal R6 strain (Hoskins et al., 2001) and pANN202312R plasmid DNA from *E. coli* (Godessart et al., 1988) were used. Chromosomal DNA from *S. pneumoniae* was prepared as described previously (Ruiz-Cruz et al., 2010). For small-scale preparations of plasmid DNA, the High Pure Plasmid Isolation Kit (Roche Applied Science) was used. The 222-bp DNA region of the R6 chromosome (coordinates 1598298–1598519) was amplified using the 1622H and 1622I primers. The 288-bp *hlyR* DNA fragment (coordinates 129–416 in Madrid et al., 2002) was amplified using the hlyR-Fw and hlyR-Rev primers. The 290-bp *hlyC* DNA fragment (coordinates 2109–2398 in Madrid et al., 2002) was amplified using the hlyC-Fw and hlyC-Rev primers.

**Table 1:** **Oligonucleotides used in this work**.

**Name**	**Sequence 5′ to 3′**
1622H	CGGATTAAACCTCTTGCAATTATACC
1622I	CAAATTCTTTAATTGTTGCTATTA
hlyR-Fw	GCCCACTGCATTGAATACTTAC
hlyR-Rev	CATACTTACCTACAGCTATAAG
hlyC-Fw	CAGATAAAGAAGAGTAGTTC
hlyC-Rev	CAAGTTTTTATTGCGCTGACTAAC
Hly Sal/Eco5	CAGACCACACCTGGAAAAAC
Hly Sal/Hind3	GGGCTTCACTGCGAAATTCA

### Radioactive labeling of DNA fragments

Oligonucleotides were radioactively labeled at the 5′-end using [γ-^32^P]ATP (PerkinElmer) and T4 polynucleotide kinase (T4 PNK; New England Biolabs). Reactions (25 μl) contained 30 pmol of oligonucleotide, 2.5 μl of 10 × kinase buffer (provided by the supplier), 50 pmol of [γ-^32^P]ATP (3000 Ci/mmol, 10 mCi/ml) and 10 units of T4 PNK. After incubation at 37°C for 30 min, additional T4 PNK (10 units) was added. Reaction mixtures were then incubated at 37°C for 30 min. The enzyme was inactivated by incubation at 65°C for 20 min. Non-incorporated nucleotide was removed using Illustra MicroSpin™ G-25 columns (GE Healthcare). The 5′-labeled oligonucleotides were used for manual sequencing and for PCR amplification to obtain double-stranded DNA fragments labeled at either the coding or the non-coding strand.

### Purification of Mga*Spn*-His and H-NS-His

Gene *mgaSpn* was engineered to encode a His-tagged Mga*Spn* protein (Mga*Spn*-His). This variant of Mga*Spn* carries six additional His residues at the C-terminal end. The procedure used to overproduce and purify Mga*Spn*-His was reported previously (Solano-Collado et al., 2012). Purified H-NS-His protein was obtained as described by Nieto et al. (1991).

### Electrophoretic mobility shift assays (EMSA)

Binding reactions (10 μl) contained 40 mM Tris-HCl, pH 7.6, 1 mM DTT, 0.4 mM EDTA, 1–2% glycerol, 50 mM NaCl, 10 mM MgCl_2_, 500 μg/ml bovine serum albumin (BSA), 2 nM of 5′-labeled DNA and varying concentrations of Mga*Spn*-His (20 to 180 nM). Reactions were incubated at ambient temperature for 20 min. Free and bound DNA forms were separated on native polyacrylamide (5%) gels (Mini-PROTEAN system, Bio-Rad) using Tris-borate-EDTA, pH 8.3, buffer (TBE). Gels were run at 100 V and ambient temperature. Labeled DNA was visualized using a Fujifilm Image Analyzer FLA-3000 and quantified using the Quantity One software (Bio-Rad).

For competitive EMSA, a 407-bp DNA fragment from the 5′ untranslated region of the *hlyR* gene was generated by PCR using primers Hly Sal/Eco5 and Hly Sal/Hind3 (Table 1). For each reaction, 50 ng of the pneumococcal 222-bp DNA and 150 ng of the 407-bp DNA (competitor DNA) were mixed with increasing concentrations of H-NS-His in binding buffer (250 mM HEPES, pH 7.4, 350 mM KCl, 5 mM EDTA, 5 mM DTT, 500 μg/ml BSA, 25% glycerol) and incubated at 37°C for 30 min. Samples (20 μl) were loaded onto native polyacrylamide (5%) gels (TBE buffer). Bands were visualized using a Gel-doc system (Bio-Rad).

### DNase I footprinting assays

In the case of H-NS-His, binding reactions (10 μl) contained 30 mM Tris-HCl, pH 7.6, 1 mM DTT, 1 mM CaCl_2_, 10 mM MgCl_2_, 100 mM NaCl, 1% glycerol, 2 nM ^32^P-labeled DNA and different concentrations of H-NS-His (2 nM to 500 nM). For Mga*Spn*-His, binding reactions (10 μl) contained 40 mM Tris-HCl, pH 7.6, 1.2 mM DTT, 0.2 mM EDTA, 1 mM CaCl_2_, 10 mM MgCl_2_, 50 mM NaCl, 1% glycerol, 500 μg/ml BSA, 2 nM ^32^P-labeled DNA and different concentrations of Mga*Spn*-His (10 to 200 nM). In all cases, after 20 min at ambient temperature, 0.03 units of DNase I (Roche Applied Science) was added and the reaction proceeded for 5 min at the same temperature. DNase I digestion was stopped by adding 1 μl of 250 mM EDTA. Then, 4 μl of loading buffer (80% formamide, 1 mM EDTA, 10 mM NaOH, 0.1% bromophenol blue and 0.1% xylene cyanol) was added. After heating at 95°C for 5 min, samples were loaded onto 8 M urea-6% polyacrylamide gels. Dideoxy-mediated chain termination sequencing reactions were run in the same gel. Labeled products were visualized using a Fujifilm Image Analyser FLA-3000. The intensity of the bands was quantified using the Quantity One software (Bio-Rad).

### *In silico* prediction of intrinsic curvature

The bendability/curvature propensity plots of the DNA fragments used in this study were calculated with the bend.it server (Vlahovicek et al., 2003; http://hydra.icgeb.trieste.it/dna/bend_it.html) as described previously (Solano-Collado et al., 2013).

## Results

### Binding of Mga*Spn*-His to the *hlyR* region of the *E. coli hly* operon

**Figure 2:**
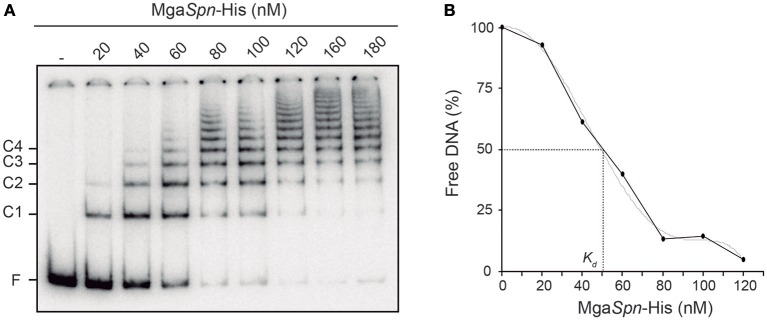
**Binding of Mga***Spn***-His to the 288-bp ***hlyR*** DNA fragment. (A)** EMSA. The ^32^P-labeled *hlyR* DNA fragment (2 nM) was incubated with increasing concentrations of Mga*Spn*-His (20 to 180 nM). Free and bound DNAs were separated by native gel electrophoresis (5% polyacrylamide). Bands corresponding to free DNA (F) and to several protein-DNA complexes (C1, C2, C3, and C4) are indicated. **(B)** Affinity of Mga*Spn*-His for the 288-bp *hlyR* DNA fragment. The autoradiograph shown in A was scanned, and the percentage of free DNA was plotted against Mga*Spn*-His concentration.

In the *E. coli* plasmid pHly152, previous DNase I footprinting assays revealed the existence of two extended H-NS binding sites upstream of the *hly* operon (sites I and II in Figure 1A) (Madrid et al., 2002). One of them (site I; coordinates 190–350 of pHly152) is included within the so-called *hlyR* regulatory sequence (Vogel et al., 1988), and was shown to play a significant role in the thermoregulation of the *hly* operon (Madrid et al., 2002). To investigate whether the pneumococcal Mga*Spn* protein is able to recognize particular regions on the H-NS binding site I, we performed EMSA and DNase I footprinting experiments. We used a His-tagged version of Mga*Spn* (Mga*Spn*-His) and a 288-bp DNA fragment (here named *hlyR*; coordinates 129–416) that contains the site I (Figure 1A). The presence of a His-tag at the C-terminal end of Mga*Spn* does not affect its DNA-binding properties (Solano-Collado et al., 2012, 2013). For EMSA, radioactively labeled DNA was incubated with increasing concentrations of Mga*Spn*-His (Figure 2A). At 20 nM of Mga*Spn*-His, free DNA and two protein-DNA complexes were detected. However, in agreement with previous results (Solano-Collado et al., 2013), as the concentration of Mga*Spn*-His was increased, complexes of lower electrophoretic mobility appeared sequentially and complexes moving faster disappeared gradually, indicating that multiple protein units bind orderly on the same DNA molecule (formation of multimeric protein-DNA complexes).

By EMSA, we estimated the affinity of Mga*Spn*-His for the 288-bp *hlyR* DNA fragment (Figure 2B). Since Mga*Spn*-His generates multiple protein-DNA complexes, the protein concentration required to bind half the DNA was determined by measuring the decrease in free DNA rather than the increase in complexes, which gives an indication of the approximate magnitude of the dissociation constant, *K*d (Carey, 1988). Such a concentration was about 50 nM. This value is similar to the apparent *K*d of Mga*Spn* for the pneumococcal 222-bp DNA fragment that harbors the *PB* activation region (Mga*Spn* binding site) (see Figure 1B) (Solano-Collado et al., 2013).

**Figure 3:**
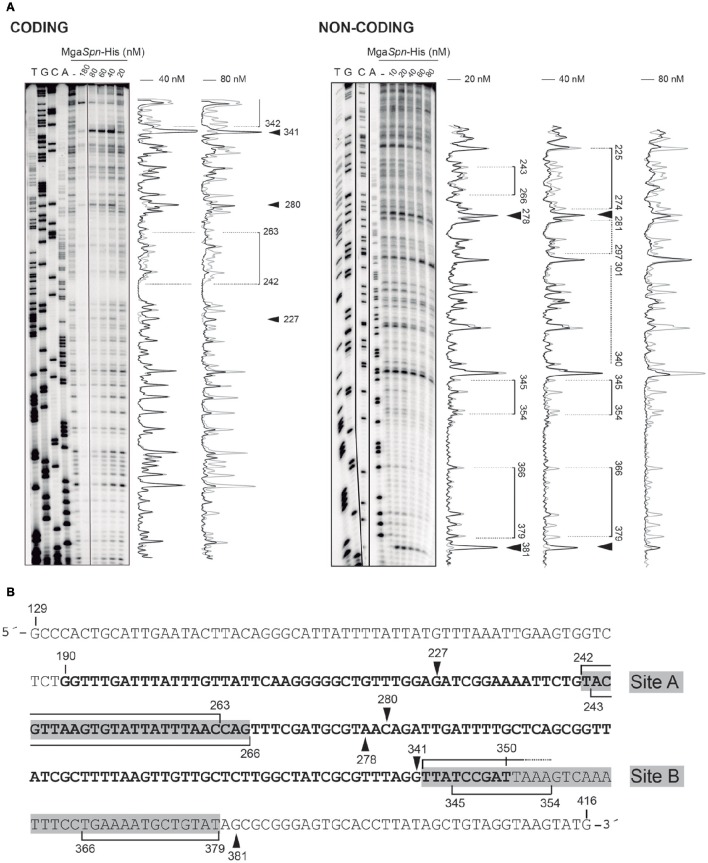
**DNase I footprints of complexes formed by Mga***Spn***-His on the 288-bp ***hlyR*** DNA fragment. (A)** The *hlyR* DNA fragment (coordinates 129–416) was radioactively labeled at the 5′-end of either the coding or the non-coding strand. Labeled DNA (2 nM) was incubated with the indicated concentrations of Mga*Spn*-His and then digested with DNase I. Dideoxy-mediated chain termination sequencing reactions were run in the same gel (lanes A, C, G, T). Densitometer scans corresponding to free DNA (gray line) and DNA with protein (black line) are shown. Brackets represent the Mga*Spn*-His protected regions. Sites more sensitive to DNase I cleavage are indicated with arrowheads. **(B)** Nucleotide sequence of the *hlyR* DNA fragment. The H-NS binding site I (coordinates 190–350) is highlighted in bold. The two sites recognized by Mga*Spn*-His (sites A and B) are marked with gray boxes. Mga*Spn*-His protected regions on either the coding or the non-coding strand (brackets) as well as sites more sensitive to DNase I cleavage (arrowheads) are indicated.

The position of Mga*Spn*-His on the *hlyR* DNA fragment was further analyzed by DNase I footprinting assays (Figure 3). The DNA fragment was labeled either at the 5′-end of the coding strand or at the 5′-end of the non-coding strand. On the coding strand and at 40 nM of Mga*Spn*-His, protections against DNase I digestion were observed at a particular region (from position 242 to 263) (Figure 3A). Diminished cleavages were also observed from position 342 onwards (no resolution in the gel). Moreover, positions 227, 280, and 341 were more sensitive to DNase I cleavage. On the non-coding strand and at 20 nM of Mga*Spn*-His, diminished cleavages were observed from 243 to 266, from 345 to 354, and from 366 to 379 (Figure 3A). Additionally, the 278 and 381 positions were more sensitive to DNase I cleavage. These results indicated that Mga*Spn*-His recognizes preferentially two sites on the *hlyR* DNA fragment (Figure 3B). One of them (site A; positions 242 to 266) is located within the H-NS binding site I, whereas the other one (site B, positions 342 to 379) is adjacent to it. On both strands and at 80 nM of Mga*Spn*-His, regions protected against DNase I digestion were observed along the DNA fragment (Figure 3A), which is consistent with the pattern of protein-DNA complexes observed by EMSA (Figure 2A).

**Figure 4:**
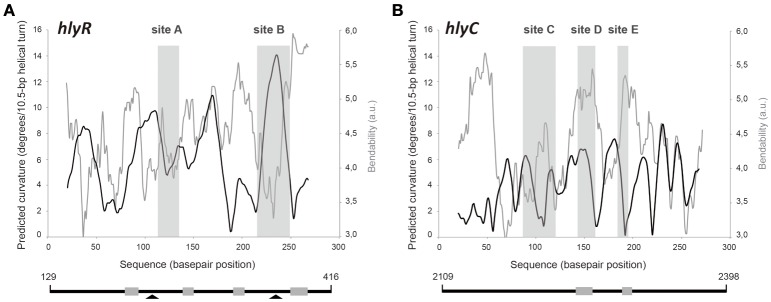
**Bendability/curvature propensity plots of the ***hlyR*** and ***hlyC*** DNA fragments according to the bend.it program (Vlahovicek et al., 2003)**. The sites recognized preferentially by Mga*Spn*-His on **(A)** the 288-bp *hlyR* DNA fragment (site A, site B), and **(B)** the 290-bp *hlyC* DNA fragment (site C, site D, and site E) are shown (gray rectangles). Lines below the plots represent the *hlyR* and *hlyC* DNA fragments. Coordinates of both DNA fragments are indicated. Gray boxes show regions of bendability that are either flanking or included into the Mga*Spn*-His binding sites. Arrowheads indicate peaks of sequence-dependent curvatures on the *hlyR* DNA fragment.

Figure 4A shows the bendability/curvature propensity plot of the 288-bp *hlyR* DNA fragment (coordinates 129–416) according to the bend.it program (Vlahovicek et al., 2003). The profile contains a peak of potential sequence-dependent curvature at position 238, just adjacent to the Mga*Spn*-His binding site A (positions 242-266). Its magnitude (9.7 degrees per helical turn) is within the values calculated for experimentally tested curved motifs (Gabrielian et al., 1997). Furthermore, the profile reveals that site A is flanked by regions of potential bendability (positions 215-222 and 271-278). The Mga*Spn*-His binding site B (positions 342-379) contains a peak of predicted curvature at position 364 (magnitude 14) which is also flanked by regions of potential bendability (positions 319-335 and 380-396). Thus, on the *hlyR* DNA fragment (see Figure 1A), Mga*Spn*-His binds preferentially to two sites (sites A and B) that are flanked by regions of potential bendability. Whereas, site A is located within the extended H-NS binding site I, site B is just adjacent to it.

### Binding of Mga*Spn*-His to the promoter region of the *E. coli hly* operon

H-NS interacts not only with the site I of plasmid pHly152 but also with the site II (coordinates 2180–2330), which is located upstream of *hlyC*, the first gene of the *hly* operon (Madrid et al., 2002) (see Figure 1A). Site II includes two of the three promoters described for the *hly* operon (Koronakis and Hughes, 1988). To analyze whether Mga*Spn*-His recognizes particular regions on the H-NS binding site II, we used a 290-bp DNA fragment (here named *hlyC*; coordinates 2109–2398) that contains the site II at internal position (Figure 1A). Radioactively labeled DNA was incubated with increasing concentrations of Mga*Spn*-His. By EMSA, we found that Mga*Spn*-His also generates multimeric complexes on the *hlyC* DNA fragment (Supplementary Figure 1). The apparent *K*d of Mga*Spn*-His for the *hlyC* DNA fragment was about 75 nM, slightly higher than for the *hlyR* DNA fragment (Figure 2B).

**Figure 5:**
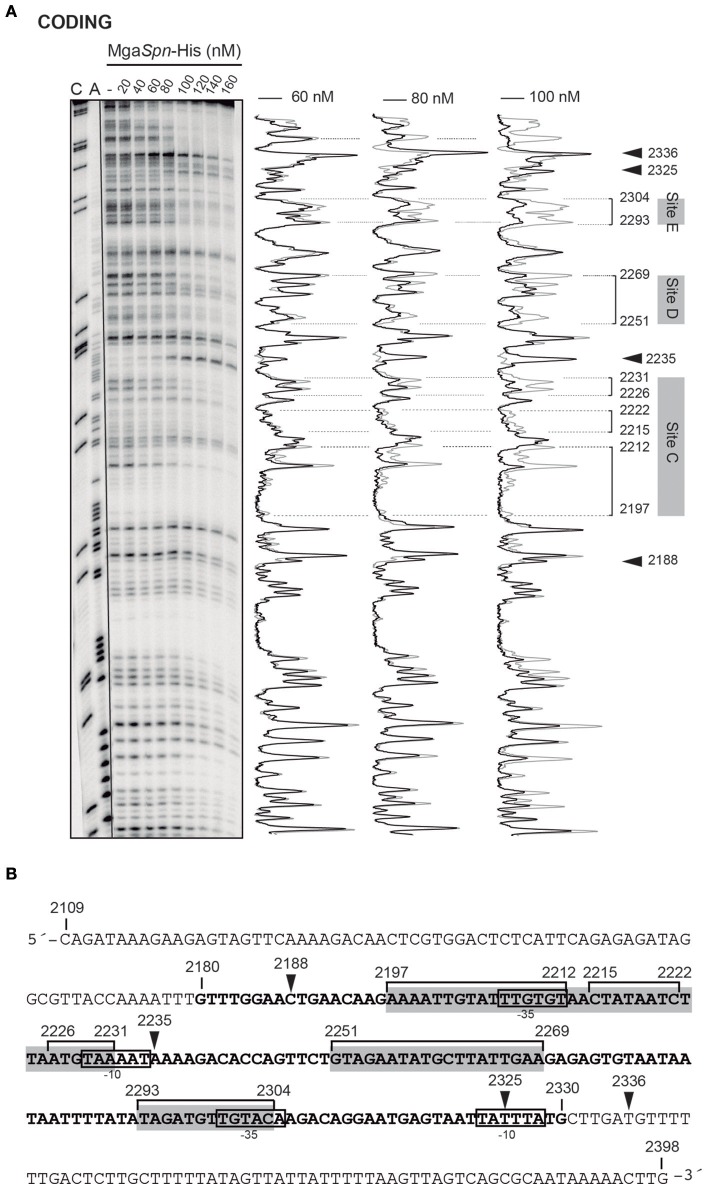
**DNase I footprints of complexes formed by Mga***Spn***-His on the 290-bp ***hlyC*** DNA fragment. (A)** The *hlyC* DNA fragment (coordinates 2109–2398) was radioactively labeled at the 5′-end of the coding strand. Labeled DNA (2 nM) was incubated with the indicated concentrations of Mga*Spn*-His and then digested with DNase I. Dideoxy-mediated chain-termination sequencing reactions were run in the same gel (lanes A, C). Densitometer scans corresponding to free DNA (gray line) and DNA with protein (black line; 60, 80, and 100 nM) are shown. The three primary sites (site C, site D and site E) recognized by Mga*Spn*-His on the *hlyC* DNA fragment are indicated. Arrowheads indicate sites more sensitive to DNase I digestion. **(B)** Nucleotide sequence of the *hlyC* DNA fragment. The −10 and −35 elements of two of the three promoters described for the *hly* operon (Koronakis and Hughes, 1988) are indicated. The H-NS binding site II (coordinates 2180–2330) identified by Madrid et al. (2002) is shown (bold letters). The three primary sites (site C, site D, and site E) recognized by Mga*Spn*-His are indicated with gray boxes. Mga*Spn*-His protected regions (brackets) as well as sites more sensitive to DNase I cleavage (arrowheads) are shown.

The position of Mga*Spn*-His on the *hlyC* DNA fragment was further analyzed by DNase I footprinting assays (Figure 5A). To this end, the 290-bp DNA fragment was labeled at the 5′-end of the coding strand. At 60 and 80 nM of Mga*Spn*-His, diminished DNase I cleavages were mainly observed from coordinate 2197 to 2231 (site C), from 2251 to 2269 (site D), and from 2293 to 2304 (site E). Moreover, positions 2188, 2235, 2325, and 2336 were more sensitive to DNase I digestion. At higher protein/DNA ratios, Mga*Spn*-mediated protections were observed along the *hlyC* DNA fragment (Figure 5A). Therefore, Mga*Spn*-His recognizes preferentially three regions within the extended H-NS binding site II (Figure 5B). Compared to the 288-bp *hlyR* DNA fragment (Figure 4A), the magnitude of the curvatures predicted in the 290-bp *hlyC* DNA fragment is slightly lower (<9 degrees per helical turn) (Figure 4B). Nevertheless, the Mga*Spn*-His binding sites D (2251-2269) and E (2293-2304) are located at regions of conspicuous bendability (positions 2248-2267 and 2296-2309, respectively).

### Binding of H-NS-His to the pneumococcal *PB* activation region

**Figure 6:**
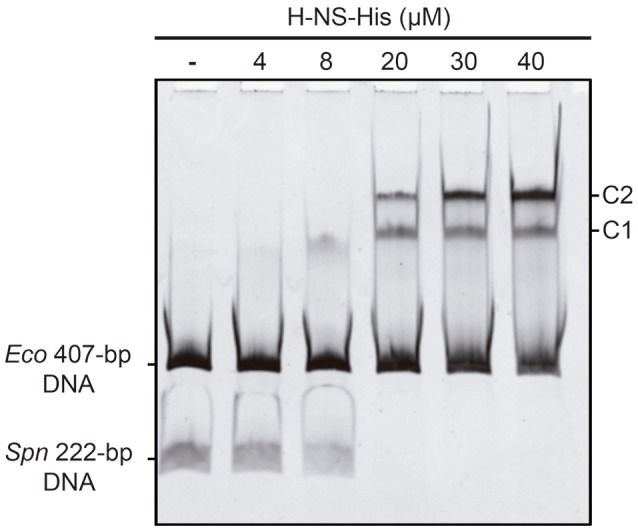
**Binding of H-NS-His to the pneumococcal 222-bp DNA fragment (competitive EMSA)**. The pneumococcal 222-bp DNA fragment (50 ng) was incubated with increasing concentrations of H-NS-His in the presence of competitor DNA (407-bp DNA fragment from the *E. coli* plasmid pHly152; 150 ng). Free and bound DNAs were separated by native gel electrophoresis (5% polyacrylamide). Bands corresponding to free DNAs and protein-DNA complexes (C1 and C2) are indicated.

**Figure 7:**
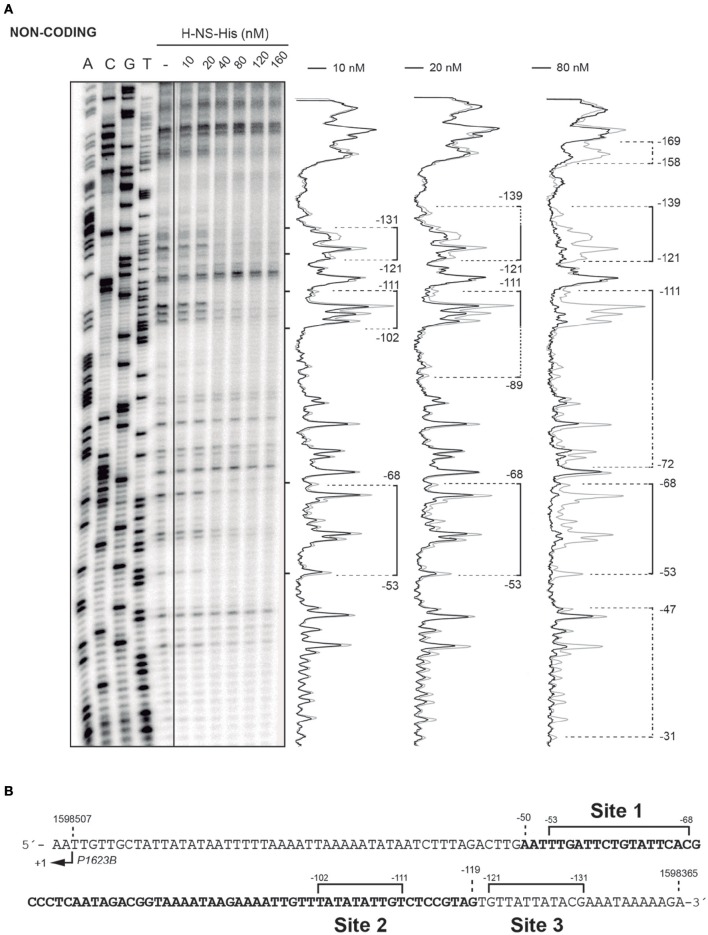
**DNase I footprints of complexes formed by H-NS-His on the 222-bp DNA fragment. (A)** The 222-bp DNA fragment of the R6 chromosome (coordinates 1598298–1598519) was radioactively labeled at the 5′-end of the non-coding strand. Labeled DNA (2 nM) was incubated with increasing concentrations of H-NS-His and then digested with DNase I. Dideoxy-mediated chain-termination sequencing reactions were run in the same gel (lanes A, C, G, T). Densitometer scans corresponding to free DNA (gray line) and DNA with protein (black line; 10, 20, and 80 nM) are shown. Brackets indicate H-NS-His binding sites. Extension of the H-NS-His mediated protections as the protein concentration increases is indicated with dotted lines. **(B)** Nucleotide sequence of the region spanning coordinates 1598365 and 1598509 of the 222-bp DNA fragment. The transcription start site from the *P1623B* promoter (+1) is shown. The *PB* activation region (positions −50 to −119), which includes the Mga*Spn* binding site, is highlighted in bold. The primary sites (sites 1, 2, and 3) recognized by H-NS-His on the 222-bp DNA fragment are indicated (brackets).

*In vivo* activation of the *P1623B* promoter requires a 70-bp region (*PB* activation region) located upstream of the promoter (from position −50 to −119) (Figure 1B). By DNase I footprinting experiments, we demonstrated previously that Mga*Spn*-His interacts with the *PB* activation region (positions −52 to −102) when it is located at internal position on a 222-bp DNA fragment (coordinates 1598298 to 1598519 of the pneumococcal R6 genome; see Figure 1B) (Solano-Collado et al., 2012). Similar results were obtained using an untagged form of the Mga*Spn* protein (Solano-Collado et al., 2013). In the present study, we analyzed whether protein H-NS-His is able to bind to the pneumococcal 222-bp DNA fragment that contains the *PB* activation region. First, we performed a competitive gel retardation assay (Figure 6). The pneumococcal 222-bp DNA fragment was mixed with a 407-bp DNA fragment (competitor DNA) from the *E. coli* plasmid pHly152, and both DNAs were incubated with increasing concentrations of H-NS-His. The 407-bp DNA fragment was reported to lack preferential binding sites for H-NS (Madrid et al., 2002). As shown in Figure 6, protein H-NS-His showed a higher affinity for the pneumococcal 222-bp DNA fragment. Next, we used DNase I footprinting to identify the sites recognized by H-NS-His. The pneumococcal 222-bp DNA fragment was radioactively labeled at the 5′-end of the non-coding strand, and then it was incubated with increasing concentrations of H-NS-His (Figure 7A). At 10 nM of H-NS-His, changes in DNase I sensitivity (diminished cleavages) were observed from positions −53 to −68 (site 1), −102 to −111 (site 2), and −121 to −131 (site 3). Whereas, site 1 and site 2 are located within the *PB* activation region, site 3 is adjacent to it (Figure 7B). At 80 nM of protein, H-NS-His mediated protections were observed along the entire DNA fragment (Figure 7A). According to predictions of intrinsic DNA curvature (Solano-Collado et al., 2013), the pneumococcal 222-bp DNA fragment contains one peak of potential curvature (magnitude 9.5, position −90) within the *PB* activation region (Figure 1B). Furthermore, there are two regions of potential bendability (from −62 to −76 and from −110 to −122) flanking such a curvature. Hence, the three sites recognized by H-NS-His on the pneumococcal 222-bp DNA fragment are adjacent to regions of potential bendability.

## Discussion

Proteins Mga*Spn* and H-NS are very different in size. They neither exhibit sequence similarity nor share a common domain structure. Mga*Spn* is a member of an emerging class of global response regulators (the Mga/AtxA family) that contain phosphoenolpyruvate **p**hospho**t**ransferase **s**ystem (PTS) **r**egulation **d**omains (PRDs) (Hammerstrom et al., 2015). Mga*Spn* is predicted to have two N-terminal helix-turn-helix DNA-binding motifs, a central PRD and a C-terminal region with amino acid similarity to the PTS protein EIIB. On the other hand, proteins in the H-NS family consist of a coiled-coil N-terminal oligomerization domain and a C-terminal DNA-binding domain. Both domains are joined via an unstructured flexible linker (reviewed by Winardhi et al., 2015). Despite these differences between Mga*Spn* and H-NS, previous studies on the Mga*Spn* transcriptional regulator suggested that it shares certain DNA-binding properties with H-NS (Solano-Collado et al., 2013).

**Figure 8:**
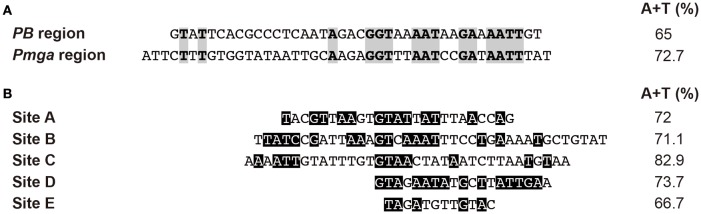
**Sites recognized by Mga***Spn***. (A)** Nucleotide sequence alignment of the *PB* activation region and the *Pmga* promoter region of the pneumococcal R6 genome. Both Mga*Spn* binding sites were defined by hydroxyl radical footprinting assays (Solano-Collado et al., 2013). Identical nucleotides are highlighted in gray boxes. **(B)** Nucleotide sequence alignment of the regions recognized by Mga*Spn*-His on the *hly* regulatory region of the *E. coli* plasmid pHly152 (this work). These Mga*Spn*-His binding sites were defined by DNase I footprinting assays. Nucleotides of such sites that are present in the *PB* activation region and/or the *Pmga* promoter region are highlighted in black boxes. The A+T content of the different sites is indicated on the right. The global A+T content of the pneumococcal R6 genome is 60.3%. By EMSA, the minimum DNA size required for Mga*Spn* binding was shown to be between 20 and 26 bp (Solano-Collado et al., 2013).

Based on *in vitro* DNA binding studies, we have proposed that Mga*Spn* regulates the expression of numerous genes by a mechanism that involves recognition of particular DNA conformations (Solano-Collado et al., 2013). By hydroxyl radical footprinting experiments, Mga*Spn* was shown to bind to two regions of the *S. pneumoniae* R6 chromosome: the *PB* activation region (positions −60 to −99 of the *P1623B* promoter) and the *Pmga* promoter region (positions −23 to +21 of the *Pmga* promoter) (Solano-Collado et al., 2013). Both Mga*Spn* binding regions share the **GGT**(A/T)(A/T)**AAT**(A/C)(A/C)**GA**(A/T)**AATT** sequence element (Figure 8A). Moreover, they contain a potential intrinsic curvature flanked by regions of bendability (Solano-Collado et al., 2013). Results presented in this work support that Mga*Spn* recognizes structural features in its DNA targets. Specifically, DNase I footprinting experiments allowed us to identify five primary sites for Mga*Spn* on the *hly* operon regulatory region of the *E. coli* plasmid pHly152. All of them are located within or adjacent to extended H-NS binding sites (Figure 1A). Moreover, four out of the five Mga*Spn* binding sites (i) have a size between 19 and 38 bp, (ii) display a high A+T content (71.1–82.9%), (iii) share short DNA sequence motifs with the two pneumococcal Mga*Spn* binding sites (Figure 8), and (iv) are either located at or flanked by regions of potential bendability (Figure 4). Considering the global A+T content (60.3%) of the pneumococcal R6 genome, these results reinforce the conclusion that Mga*Spn*, like H-NS, has a preference for AT-rich DNA sites. Most likely, a preference for AT-rich DNA regions rather than for specific DNA sequences is a general feature of the global regulators that constitute the Mga/AtxA family. Sequence alignments of all established Mga binding regions revealed that they exhibit only 13.4% identity. Furthermore, a mutational analysis in some target promoters indicated that Mga binds to DNA in a promoter-specific manner (Hause and McIver, 2012). In the case of AtxA, sequence similarities in its target promoters are not apparent, and it has been shown that the promoter regions of several target genes are intrinsically curved (Hadjifrangiskou and Koehler, 2008).

Proteins of the H-NS family have a high degree of sequence similarity in the DNA-binding domain, as it is the case of H-NS and Ler (Shindo et al., 1995; Cordeiro et al., 2011). The three-dimensional structure of a complex between the DNA-binding domain of Ler and a 15-mer DNA duplex has been solved (Cordeiro et al., 2011). This structure revealed that the DNA-binding domain of Ler does not participate in base-specific contacts but recognizes specific structural features in the DNA minor groove. Thus, Ler, and likely other members of the H-NS family, recognizes specific DNA shapes. By DNase I footprinting experiments, we have found that H-NS recognizes three particular sites on the regulatory region of the pneumococcal *P1623B* promoter (Figure 1B). The three sites, ranging in size from 10 to 16 bp, are adjacent to regions of potential bendability, which agrees with the preference of H-NS for AT-rich DNA regions. Moreover, two out of the three H-NS binding sites occur in the *PB* activation region, which includes an Mga*Spn* binding site. Hence, the regulatory region of the pneumococcal *P1623B* promoter contains structural motifs that are recognized by H-NS.

In conclusion, two categories of protein-DNA interactions, namely those in which the protein recognizes unique chemical signatures of the DNA bases (base readout), and those in which the protein recognizes a sequence-dependent DNA shape (shape readout) were defined by Rohs et al. (2010). Our present work suggests that two unrelated DNA-binding proteins from phylogenetically distant bacteria are able to recognize similar structural characteristics in their DNA targets. It is intriguing that unrelated bacterial species have evolved to encode proteins that seem to use a similar strategy to regulate the expression of a number of genes (silencing or activations). We take this as an indication of a successful strategy for proteins recognizing DNA regions that show intrinsic bendability/flexibility.

## Author contributions

VS and MH performed laboratory work. VS, MH, ME, AJ, and AB designed the study, performed data analysis and wrote the manuscript. All authors read and approved the final manuscript.

### Conflict of interest statement

The authors declare that the research was conducted in the absence of any commercial or financial relationships that could be construed as a potential conflict of interest.

## Supplementary material

Supplementary Figure 1 **Binding of Mga***Spn***-His to the 290-bp ***hlyC*** DNA fragment. (A)** EMSA. The ^32^P-labeled *hlyC* DNA fragment (2 nM) was incubated with increasing concentrations of Mga*Spn*-His (20 to 180 nM). Free and bound DNAs were separated by native gel electrophoresis (5% polyacrylamide). Bands corresponding to free DNA (F) and to several protein-DNA complexes (C1, C2, C3, and C4) are indicated. **(B)** Affinity of Mga*Spn*-His for the 290-bp *hlyC* DNA fragment. The autoradiograph shown in A was scanned, and the percentage of free DNA was plotted against Mga*Spn*-His concentration.

## Abbreviations

BSA: bovine serum albumin
EMSA: electrophoretic mobility shift assays
*hly*: hemolysin
PCR: polymerase chain reaction
T4 PNK: T4 polynucleotide kinase.
