# Supplementary figures and images for: MgaSpn and H-NS: Two Unrelated Global Regulators with Similar DNA-Binding Properties
Source: Front Mol Biosci. 2016 Sep 29;3:60. doi: 10.3389/fmolb.2016.00060 (PMC5040716; doi:10.3389/fmolb.2016.00060)

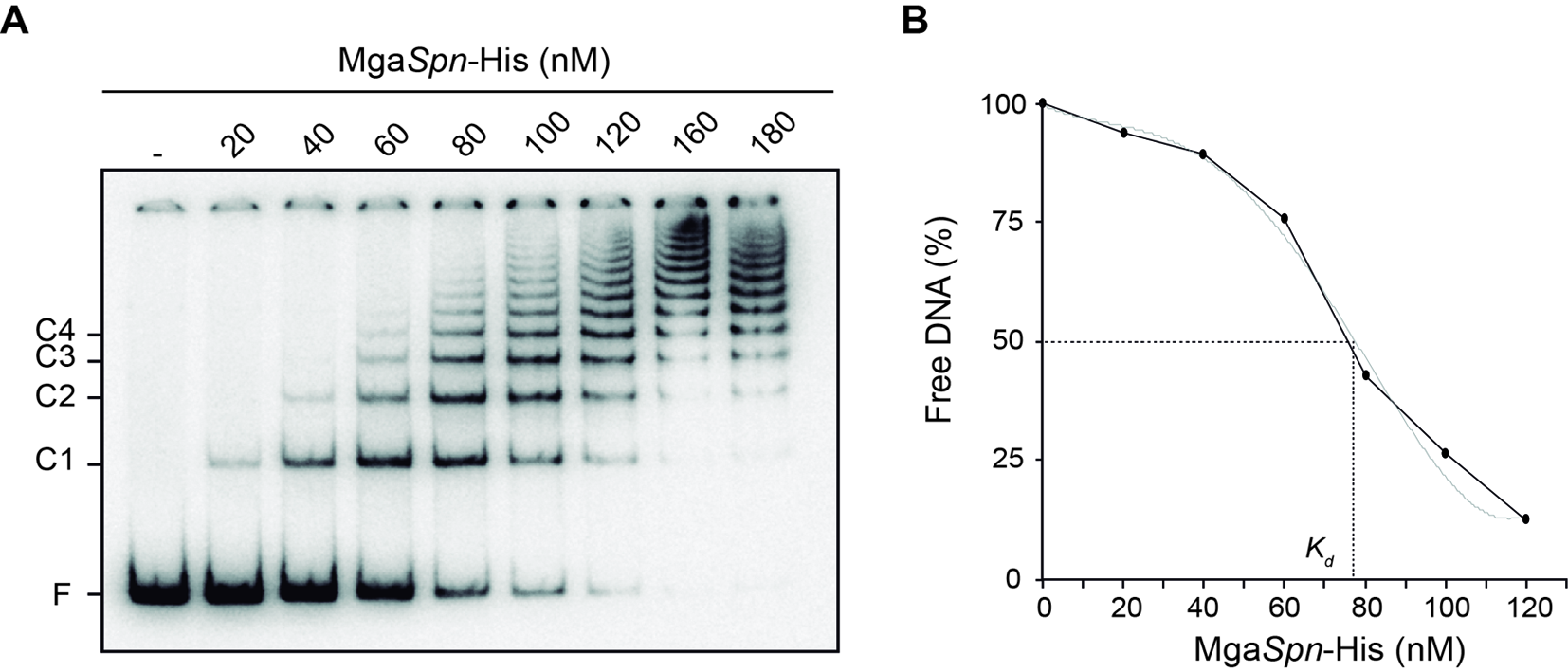

Supplement: Supplementary Figure 1 — Binding of MgaSpn-His to the 290-bp hlyC DNA fragment. (A) EMSA. The 32P-labeled hlyC DNA fragment (2 nM) was incubated with increasing concentrations of MgaSpn-His (20 to 180 nM). Free and bound DNAs were separated by native gel electrophoresis (5% polyacrylamide). Bands corresponding to free DNA (F) and to several protein-DNA complexes (C1, C2, C3, and C4) are indicated. (B) Affinity of MgaSpn-His for the 290-bp hlyC DNA fragment. The autoradiograph shown in A was scanned, and the percentage of free DNA was plotted against MgaSpn-His concentration. [file Image1.TIF]
